# Application of Carboxymethyl Cellulose and Glycerol Monostearate as Binder Agents for Protein Powder Production from Honey Bee Brood Using Foam-Mat Drying Technique

**DOI:** 10.3390/foods13142265

**Published:** 2024-07-18

**Authors:** Supakit Chaipoot, Rewat Phongphisutthinant, Pairote Wiriyacharee, Gochakorn Kanthakat, Worachai Wongwatcharayothin, Chalermkwan Somjai, Khanchai Danmek, Bajaree Chuttong

**Affiliations:** 1 Multidisciplinary Research Institute, Chiang Mai University, Chiang Mai 50200, Thailand; supakit.ch@cmu.ac.th; 2 Research Center of Microbial Diversity and Sustainable Utilization, Faculty of Science, Chiang Mai University, Chiang Mai 50200, Thailand; pairote.w@cmu.ac.th; 3 Faculty of Agro-Industry, Chiang Mai University, Chiang Mai 50100, Thailand; gochakorn.g@gmail.com (G.K.); worachai.fst@gmail.com (W.W.); 4 Processing and Product Development Factory, The Royal Project Foundation, Chiang Mai 50100, Thailand; chalermkwansomjai@hotmail.com; 5 School of Agriculture and Natural Resources, University of Phayao, Phayao 56000, Thailand; khanchai.da@up.ac.th; 6 Meliponini and Apini Research Laboratory, Department of Entomology and Plant Pathology, Faculty of Agriculture, Chiang Mai University, Chiang Mai 50200, Thailand

**Keywords:** bee brood, foam-mat drying technique, binder agents, protein powder

## Abstract

This study investigates the development of protein powder from honey bee drone broods using foam-mat drying, a scalable method suitable for community enterprises, as well as the preservation of bee broods as a food ingredient. Initially, honey bee broods were pre-treated by boiling and steaming, with steamed bee brood (S_BB) showing the highest protein content (44.71 g/100 g dry basis). A factorial design optimized the powder formulation through the foam-mat drying process, incorporating varying concentrations of S_BB, glycerol monostearate (GMS), and carboxymethyl cellulose (CMC). The physicochemical properties of the resulting powder, including yield, color spaces, water activity, solubility, protein content, and total amino acids, were evaluated. The results showed that foam-mat drying produced a stable protein powder. The binders (CMC and GMS) increased the powder’s yield and lightness but negatively affected the hue angle (yellow-brown), protein content, and amino acid content. The optimal quantities of the three variables (S_BB, GMS, and CMC) were determined to be 30 g, 6 g, and 1.5 g, or 80%, 16%, and 4%, respectively. Under this formulation, the protein powder exhibited a protein content of 19.89 g/100 g. This research highlights the potential of bee brood protein powder as a sustainable and nutritious alternative protein source, enhancing food diversification and security.

## 1. Introduction

Honey bee brood, consisting of the larvae and pupae stages of honey bees, is an underutilized yet highly nutritious resource. It exhibits a rich profile of proteins, essential amino acids, fatty acids, vitamins, and minerals, which positions it as a potentially valuable food source to address global nutritional needs and food security challenges [1,2]. Despite its impressive nutritional benefits, the consumption of bee brood remains largely confined to specific cultural contexts. In parts of Asia and Africa, it is traditionally regarded as a delicacy, often consumed during specific festivals or as part of traditional food [1,2,3]. The growing interest in edible insects as sustainable protein sources provides a unique opportunity to explore the broader use of bee brood in contemporary diets. Edible insects, including bee brood, are increasingly recognized for their environmental benefits, such as lower greenhouse gas emissions, reduced land and water use, and high feed conversion efficiency compared to traditional livestock [4,5,6]. This shift towards alternative protein sources is crucial as the global population continues to rise and the demand for sustainable and nutritious food sources becomes more pressing.

Protein powders derived from insect sources have gained significant attention due to their environmental benefits and high nutritional value. These powders can serve as sustainable alternatives to conventional protein sources, such as meat and dairy, helping to meet the rising global demand for protein [7,8]. The production of insect protein powders is not only more environmentally friendly but also offers adaptability in their use, from incorporation into baked goods and snacks to functional ingredients in nutritional supplements.

Foam-mat drying involves whipping the product into a foam before drying, which aids in producing a fine, uniform, and easily reconstituted powder. This method has been successfully used for drying various food products, maintaining their nutritional qualities while improving shelf life and convenience [9,10]. Various binders such as gums, proteins (egg albumen, soy protein isolate, whey protein concentrate, and gelatin), methylcellulose, carboxymethyl cellulose (CMC), and glycerol monostearate (GMS) are commonly used as foaming agents to form stabilized foams during the agitation and drying process through foam-mat drying under conventional conditions. This technique provides highly effective production with low cost and simple processing steps compared to other drying techniques such as drum drying, spray drying, vacuum drying, and freeze drying [10,11]. An essential attribute of stable foam production is its ability to produce high-quality foam-mat powder. GMS acts as a lipid-based foaming agent, while CMC serves as a foaming stabilizer. These agents retain the gas–liquid foam structure by reducing the surface tension of the thin liquid film between the interfaces, thereby delaying coalescence during thermal and airflow dehydration [12]. Several studies have investigated powder production through the foam-mat drying process using various fruits, vegetables, cereals, marine products (such as shrimp), milk, eggs, and plant-based products [10,13]. However, there is a lack of research on the use of insect proteins with the foam-mat drying technique.

This study focuses on the development of a protein powder from bee brood using foam-mat drying, a method known for preserving nutritional integrity and enhancing storage stability and usability. The use of bee brood as a food ingredient has gained significant interest due to its nutritional value [1,2,3,4,5,6]. However, there is a noticeable gap in the research concerning effective preservation methods to maintain its quality over time. Initially, a suitable pre-treatment procedure for protein extraction from bee brood was investigated. This procedure was then selected for further processing of bee brood protein powder using the foam-mat drying technique. The research subsequently focused on formulating the pre-treated bee brood sample with GMS and CMC as binder agents by varying their concentrations. A factorial design was employed to optimize the foam-mat dried protein powder formulation by evaluating the physicochemical properties of the resulting powders, including yield, color, water activity, protein content, and solubility. The optimized formulation was replicated to obtain the final bee brood protein powder, which was measured for its physicochemical properties and powder particle morphology. Our study is unique in that it investigates preservation techniques to fill a crucial gap in current knowledge. Previous studies have primarily focused on the nutritional aspects of bee brood, but none have thoroughly explored how to best preserve it for extended use. Thus, the findings from this research could be valuable for beekeepers and the food industry, particularly those interested in using bee brood as a food ingredient. Foam-mat drying is a scalable method suitable for community enterprises, enabling the production of high-quality protein powder from bee brood. Assessing its nutritional and functional properties promotes the sustainable use of nutrient-rich bee brood in the global food industry.

## 2. Materials and Methods

### 2.1. Materials and Chemicals

Honey bee (*Apis mellifera* L.) drone broods (larva to pre-pupa stages) from an apiary in Phare Province, Thailand, were used as raw material in this study. Glycerol monostearate (GMS) and carboxymethyl cellulose (CMC) were purchased from Union Science Co., Ltd. (Chiang Mai, Thailand). The chemical substances, reagents, and standards applied in this research included boric acid (RCI Labscan, Bangkok, Thailand), ethanol (RCI Labscan, Thailand), hexane (RCI Labscan, Thailand), methanol (AR Grade) (RCI Labscan, Thailand), N-acetyl-L-cysteine (Merck, Darmstadt, Germany), octanoic acid (Merck, Germany), O-phthalaldehyde (OPA) (Sigma-Aldrich, Tokyo, Japan), perchloric acid 70% (QRec, Auckland, New Zealand), potassium sulfate (RCI Labscan, Thailand), sodium carbonate (QRec, New Zealand), sodium citrate tribasic dihydrate (RCI Labscan, Thailand), sodium hydroxide (RCI Labscan, Thailand), sodium hypochlorite solution (4–6%) (Loba Chemie, Mumbai, India), and trichloroacetic acid (TCA 99%) (Merck, Germany). A set of analytical amino acid standard mixtures, consisting of 17 amino acids, was obtained from Wako Pure Chemical Corporation (Osaka, Japan). All dilutions and solutions were prepared with demineralized water produced from a water purification system (Zeneer UP 900, Seoul, Republic of Korea).

### 2.2. Bee Brood Preparation

Two distinct cooking methods, boiling and steaming, were employed in the preparation of bee brood for protein extraction. In the boiling process, bee brood was immersed in hot water (95 °C) for 15 min and subsequently dried at 60 °C for 18 h using a hot air dryer (Memmert: Model UM 500, Schwabach, Germany). For the steaming process, bee brood was subjected to steam (approximately 100 °C) for 15 min, following the same procedure as the boiling process. Both the boiled bee brood (B_BB) and steamed bee brood (S_BB) samples were analyzed for proximate and amino composition. Additionally, raw bee brood was dried, tested, and served as a control sample. The chosen cooking method for bee brood preparation was selected for further investigation in the subsequent study.

### 2.3. Experimental Design for Bee Brood Protein Powder Formulation and Foam-Mat Drying Condition

A suitable steam cooking method was employed to prepare bee brood for the production of protein powder. The steamed bee brood (S_BB), without undergoing a drying process, was mixed with water in different weight ratios of S_BB to water (*w*/*w*): 10:90, 20:80, and 30:70, using a hand blender (800W, Philips, Bangkok, Thailand). Each mixture was blended into a slurry-like consistency. Subsequently, the mixtures were gradually stirred and heated in a stainless-steel double boiler pot until they reached 70 °C, after which binder agents were added to aid in dissolution. The foam-mat drying process involved the addition of binder agents (GMS, and CMC), which formed a semi-liquid. The complete blend was then agitated for 10 min using a bowl-lift stand mixer (KitchenAid heavy-duty stand mixer 5KPM5, Greenville, OH, USA) to generate foam. The resulting foam sample was placed on an aluminum tray and subjected to drying in a hot air oven at 70 °C for approximately 3 h or until the moisture content did not exceed 4%. Subsequently, the dried samples underwent grinding to achieve a powder form.

The investigation considered three variable factors: S_BB in the range of 10–30 g, GMS in the range of 6–14 g, and CMC in the range of 0.5–1.5 g. The formulation of bee brood powder was developed through a factorial design (2^3^) with three replications at the center points, as outlined in Table 1. Subsequently, eleven formulations of bee brood protein underwent foam-mat drying production to generate protein powder. All experimental runs were analyzed based on physicochemical characteristics and powder qualities.

### 2.4. Nutritional and Amino Acids Component of Bee Brood

#### 2.4.1. Proximate Analysis

Proximate analysis, including the determination of moisture content, crude protein content using the combustion method (nitrogen-to-protein conversion factor of 6.25) [1], crude fiber, fat content, and carbohydrate, was conducted on three samples: B_BB, S_BB, and the control (raw bee brood). The procedures outlined by AOAC [14] were followed for these analyses. Each sample underwent analysis in triplicate, and the average values were subsequently calculated. Furthermore, the protein content of the bee brood powders was subjected to analysis.

#### 2.4.2. Amino Acids Determination by HPLC

The analysis of 17 amino acids was carried out using a post-column reaction method as outlined by Somjai et al. [15]. The analytical setup included a Shim-pack AMINO-NA column (100 mm length × 6.0 mm I.D., with 5 μm particle size, P/N: 228-18837-91, Shimadzu, Kyoto, Japan) with a prominence RF-20A fluorescence detector (Shimadzu, Japan). Mobile phases A, B, and C, identified as sodium citrate buffers with pH values of 3.23 (A) and 10.0 (B), and C comprising an aqueous solution of 0.2 M NaOH, were employed. Reaction reagents for pre-column derivatization of amino acids were created from N-Acetyl-L-cysteine and OPA. Operating conditions encompassed a flow rate of 0.4 mL/min, a column oven temperature set at 60 °C, and a sample injection volume of 10 µL. All samples in this study were evaluated for the quantity of amino acids.

### 2.5. Bee Brood Protein Powder Qualities

#### 2.5.1. Powder Yield

The powder yield was determined by calculating the ratio of the mass of the final powder obtained after hot air drying to the initial mass of the starting mixture, including both GMS and CMC [16], as expressed in Equation (1):

Powder yield (%) = (Weight of powder/Weight of mixture) × 100(1)

#### 2.5.2. Color Space Analysis

The color characteristics (L*, C*, h) of bee brood protein powder were assessed with a CR-400 colorimeter (Konica, Minolta, Tokyo, Japan). In this context, L* corresponds to brightness, C* denotes chroma, and h signifies the hue angle in the color space analysis.

#### 2.5.3. Water Activity (aw)

The water activities were determined by a water activity meter (model AWC200, Novasina, Lachen, Switzerland).

#### 2.5.4. Solubility

The solubility of bee brood protein powder was evaluated following the method described by Vidovic et al. [17]. A precisely measured amount of 2.500 g of the powder was dissolved in 30 mL of deionized water. The obtaining mixture was centrifuged at 3000 rpm for 15 min, and only the supernatant was retained for drying in a hot air oven at 105 °C for 3 h. The soluble ability was then calculated using Equation (2):

Solubility (%) = [(weight of supernatant after drying − weight of powder)] × 100(2)

#### 2.5.5. Bulk and Tapped Densities of Powder

Following the methods of Kamali et al. [11] and Omidi et al. [18], approximately 2 g of optimized foam-mat dried powder was placed into a 10 mL graduated cylinder without tapping to measure bulk density. For tapped density, the same cylinder was then tapped 10 times on a soft fabric from a height of 15 cm. Bulk and tapped densities were calculated using Equations (3) and (4):

Bulk density (g/cm^3^) = (Mass of powder/Volume of powder) (3)

Tapped density (g/cm^3^) = (Mass of powder/Final tapped volume) (4)

#### 2.5.6. Scanning Electron Microscopy (SEM)

The particle morphology of the optimized foam-mat dried powder was examined using a JSM-IT100 SEM (JEOL, Tokyo, Japan). The powder was placed on a metal stub, coated with gold, and the micrographs were obtained under SEM at an accelerating voltage of 5 kV with scales of 20, 50, and 100 µm.

### 2.6. Statistical Analysis

The results derived from the central composite design (CCD) experiment were analyzed using Design Expert (Version 12.0, Statease Inc., Minneapolis, MN, USA). Various combinations of responses, as described in the experimental design, were evaluated. An analysis of variance (ANOVA) was performed to confirm the adequacy of the suggested model, verify the significance of each coefficient, and assess the interaction strength of each parameter for the bee brood powder formulation. The lack of fit was not significant (*p* > 0.05) for the evaluated variables, indicating that the model accurately fit the data and was suitable for predicting the relevant responses. The optimal formulation for bee brood protein powder using the foam-mat drying technique was determined through response surface analysis and regression equations, with a confidence level of 95% used to assess the significance of the differences.

## 3. Results and Discussion

### 3.1. Nutritional and Amino Acid Compositions of Different Methods for Bee Brood Preparation

**Figure 1:**
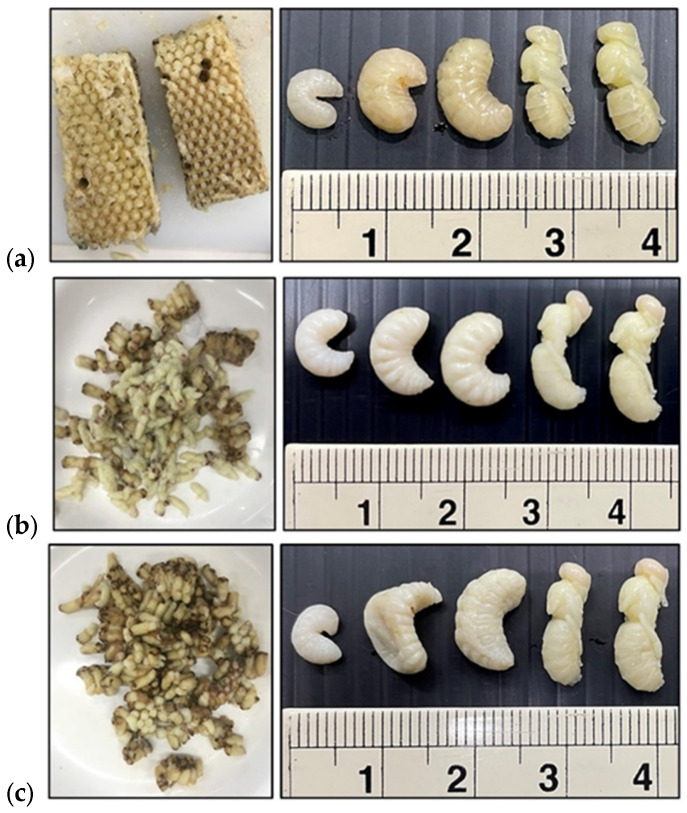
(**a**) Raw bee brood, (**b**) boiled bee brood (B_BB), (**c**) steamed bee brood (S_BB).

Three pre-treatment methods of bee brood samples are illustrated in Figure 1, and the proximate analysis, presented in Table 2, indicates that all dried bee brood samples displayed moisture content within the range of 3.31–4.61%. Additionally, the samples exhibited protein, carbohydrate, and lipid values ranging from 39.18 to 44.71 g/100 g, 26.92 to 32.81 g/100 g, and 25.32 to 29.83 g/100 g, respectively. The dried bee brood also contained minor amounts of ash and fiber, with values ranging from 3.35 to 4.27 g/100 g and 2.0 to 2.5 g/100 g, respectively. The S_BB significantly yielded the highest protein content compared to the other samples, while the B_BB exhibited the highest levels of carbohydrates and ash (*p* ≤ 0.05). Nevertheless, the levels of lipids and ash were observed to be at their maximum in the raw bee broods.

Various pre-treatment methods, including steaming, blanching, boiling, drying, frying, stewing, curing, roasting, and others, have been employed for edible insect larvae. These methods serve the dual purpose of decontamination control and enhancement of nutritional and sensory qualities, as suggested by Melgar–Lalanne et al. [19] and Ojha et al. [20]. The preparation of bee broods through boiling or steaming resulted in a decrease in lipid content, possibly attributed to the melting of lipid globules into the boiling water during thermal treatment [21]. On the other hand, the steaming process demonstrated the highest protein content, reaching 44.71 g/100 g of the dry sample, aligning with findings by Djikeng et al. [22], who observed an increase in protein content in snail meat after the steaming process compared to raw snail samples. This phenomenon might be attributed to the heat and processing time, which facilitated the release of certain protein molecules, enabling them to form stronger protein structures that are less prone to leaching into water during the steaming process. In contrast, the boiling treatment of bee larvae might induce protein denaturation and dissolution in boiling water, resulting in a reduction of nitrogen content in amides and amines due to the intense thermal treatment [21,23]. Furthermore, the ash and fiber components might undergo alterations, either increasing or decreasing, depending on the pre-treatment method used and the insect species considered [21].

The composition of seventeen amino acids in different methods of bee brood preparation was determined using HPLC, as seen in Table 3. Glycine was the predominant amino acid in bee brood, ranging from 23.87 to 45.29 mg/100 g on a dry basis, with B_BB yielding the highest levels of this amino acid type compared to other samples. Notably, raw bee brood exhibited only fourteen types of amino acids, with aspartic acid, threonine, and arginine being undetected. The levels of certain amino acids, such as serine, proline, alanine, valine, methionine, isoleucine, leucine, and phenylalanine, were significantly reduced after pretreatment involving a heating process. Conversely, thermal treatment was observed to increase specific amino acids, including aspartic acid, threonine, glycine, cysteine, tyrosine, histidine, lysine, and arginine.

The boiling process resulted in the highest total amino acid content in bee broods, followed by raw and steamed bee broods. The higher temperatures used during boiling, compared to steaming, lead to protein denaturation, affecting the retention of amino acids and proteolysis. This process can potentially destroy or modify amino acid quantities [19,24]. In addition, the Maillard reaction may occur, forming complex structures of sugar–amino acids [25]. Nevertheless, the steaming process was selected as the preferred method for the pre-treatment of bee brood in preparation for the subsequent experiment involving the production of protein powder using the foam-mat drying technique. This method was chosen because it only slightly affected protein structure denaturation and maintained the protein content when the proximate analysis was performed. This is advantageous for reducing protein loss during the heating phase of protein powder production through foam-mat drying.

### 3.2. Influence of Bee Brood Quantity and Binder Agents on the Characteristics of Dried Foam-Mat Bee Brood Protein Powder

All average data for the physicochemical responses of each run of bee brood protein powder are presented in Table 4. The obtained yield of bee brood protein powder ranged from 5.82% to 11.30%, with water activity levels varying between 0.229 and 0.441. These amounts of free water were significantly low, mitigating the risk of microbial growth and preventing chemical and biological changes, thereby ensuring the stability of the powder shelf-life [26,27]. The protein powders exhibited a light brown color in the L* (lightness), C* (chroma), and h (hue angle) color spaces, with values falling within the ranges of 64.79–77.66, 7.71–13.93, and 59.47–66.43, respectively. In addition, the protein and total amino acid contents (sum of 17 amino acids) were examined for each bee brood protein powder, revealing amounts ranging from 5.06 to 20.35 g/100 g and 6.87 to 22.13 mg/g, respectively. The solubility of each protein powder sample was also tested, with values ranging from 8.12% to 27.72%.

The analysis of variance (ANOVA, *p* < 0.05) for the various independent factors (ratios of S_BB, GMS, and CMC) on the responses and the significant equation models for each response are presented in Table 5. The yield and L* values of bee brood protein powder were well-suited for a two-factor interaction (2FI) model, while the hue angle, protein content, and amino acid content were fitted with a linear model. In contrast, these ratios showed non-significant effects on the characteristics of C* value, aw, and solubility.

**Figure 2:**
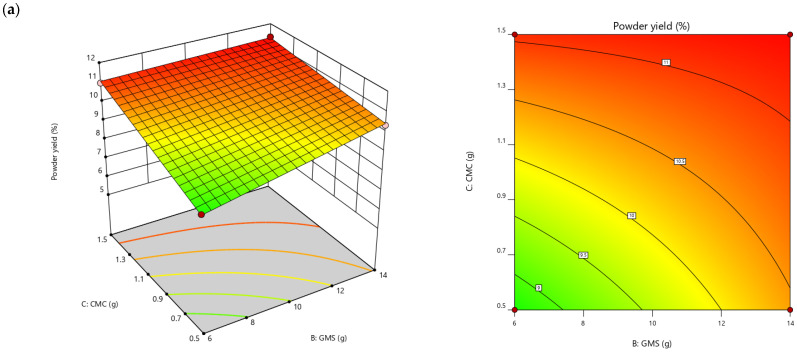

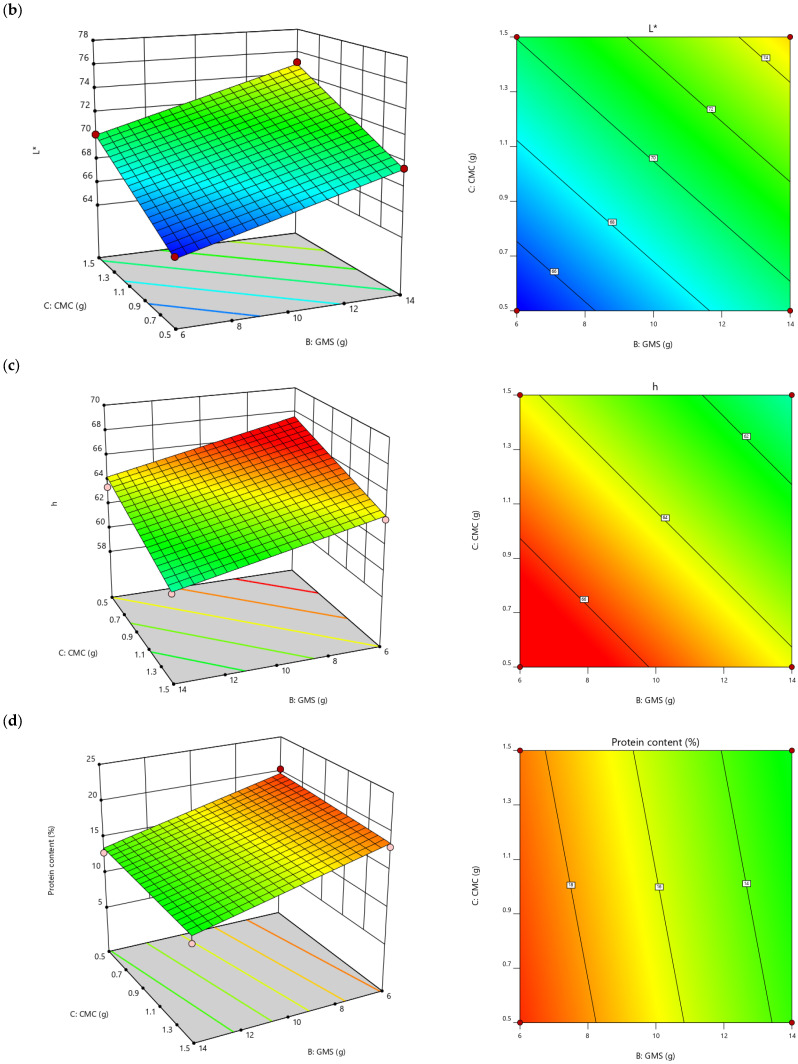

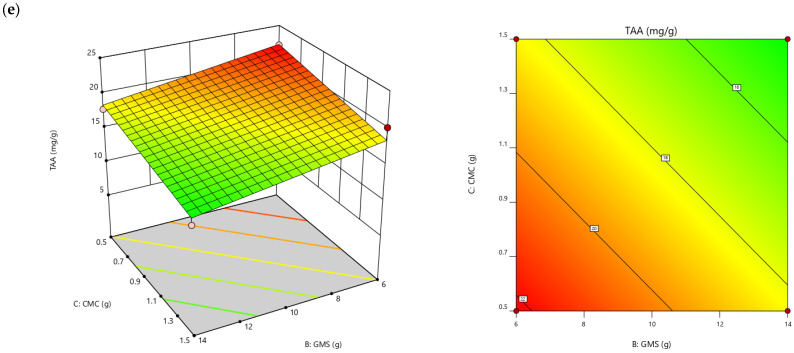
Three-dimensional (3D) surface and contour plots illustrate the interaction effects of the GMS, and CMC ratios with a fixed S_BB content (30 g) on the physicochemical responses of bee brood protein powder production, including (**a**) powder yield, (**b**) L* (lightness value), (**c**) h (hue angle), (**d**) protein content, and (**e**) TAA (total amino acids, representing the sum of 17 amino acids).

The effect of ingredient variables on powder yield demonstrated an increase with higher solids addition for powder production. Simultaneously, an increase in S_BB content resulted in a reduction in lightness value. Furthermore, higher S_BB content contributed to an increase in color hue, protein content, and TAA, while an increase in the binder agents (CMC and GMS) was associated with a decrease in these observed responses, as seen in Figure 2. A similar trend of increased powder yield with a rise in the concentration of binder agents was observed, as reported by Thakur et al. [16]. In their study, different concentrations of GMS and CMC were investigated to produce foam-mat-dried guava powder, with approximately 2% of GMS yielding about 14.7%. Additionally, Bhardwaj et al. [28] reported a 10.7% powder yield for papaya leaf foam-mat powder with 3% CMC. A linear increase in lightness was observed in direct proportion to the concentration of the binder agent. This effect can be attributed to the incorporation of the binder powder along with foamed air during the foam preparation process, resulting in lighter-colored foam-dried powders [29].

### 3.3. Optimal Formulation and Physicochemical Characterization of Dried Foam-Mat Bee Brood Protein Powder

Response surface methodology was applied to investigate the optimal formulation for achieving maximum levels of powder yield, protein, and amino acid content in dried foam-mat bee brood protein powder. This included constraining the L* and h color values within the range obtained from the experimental runs. The numerically predicted solutions, derived from the design expert program, identified the optimal quantities of three variables (S_BB, GMS, and CMC), which were 30 g of S_BB, 6 g of GMS, and 1.5 g of CMC, corresponding to percentages of 80%, 16%, and 4% of the total solid content in the formulation, respectively. These values achieved a desirability score of 0.862, with a predicted protein content of 18.58 g/100 g for this formulation. When this formulation was replicated, the resulting foam-mat bee brood powder revealed a protein content of 19.89 ± 1.13 g/100 g. The physical properties of the powder included color spaces (L*, C*, h), water activity, and solubility, with values of 71.66, 63.45, 11.8, 0.230, and 17.51%, respectively. Moreover, the bulk and tapped densities of this foam-mat powder were found to be 0.461 and 0.488 g/cm^3^, respectively. Bulk and tapped densities are closely associated with the rehydration properties, packing structure, compression characteristics, and particle arrangement of the powder. Consequently, powders with a higher bulk density can be stored in smaller packages, which is advantageous for shipping and marketing [11,18].

**Figure 3:**
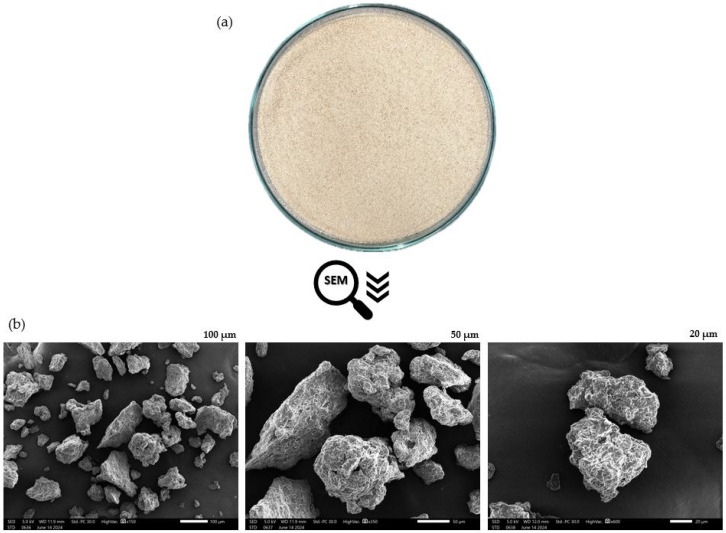
(**a**) Dried foam-mat bee brood protein powder, (**b**) SEM micrographs of dried foam-mat bee brood protein powder from the optimized formulation (scale bars: 100, 50, and 20 µm).

Additionally, the foam-mat bee brood protein powder exhibited a yellow-brown color with a fine powder texture, as depicted in Figure 3. SEM micrographs of the powder revealed surface particles characterized by rough spheres, flaky structures, and non-uniform cavities, likely due to air incorporation during hot air drying. The microstructure or particle morphology is crucial in determining the powder’s application, as it affects both the rehydration ability and the appearance of the reconstituted product [30]. Omidi et al. [18] and Bahriye et al. [31] conducted similar studies on the particle structure of foam-mat powders, observing irregular shapes and unevenness across their samples. They concluded that variations in drying conditions, such as air temperature and foam thickness, had minimal impact on the microstructural properties and porosity of the foam-mat powder particles [31].

## 4. Conclusions

The steam-water method proved to be suitable for the pre-treatment of bee brood, as it demonstrated the highest protein content with minimal loss of amino acid composition. Consequently, the steaming process was chosen as the preferred method for preparing bee brood prior to producing protein powder using the foam-mat drying technique. Binder agents, such as CMC and GMS, were incorporated to create a foam mixture with the bee brood before hot air drying. The optimal quantities of steamed bee brood, GMS, and CMC were determined to be 30 g, 6 g, and 1.5 g, corresponding to 80%, 16%, and 4% of the total solid content in the formulation, respectively. These proportions were predicted to result in the maximum yield of dried foam-mat powder with high protein content. The replicated production of foam-mat bee brood powder using the optimized formulation yielded a protein content of 19.89 g/100 g. The powder also exhibited physical properties such as color spaces (L*, C*, h), water activity, and solubility, with values of 71.66, 63.45, 11.8, 0.230, and 17.51%, respectively. Thus, this protein powder could serve as an ingredient in food products or as a suitable alternative protein source for consumers who eat edible insects, and it also offers an opportunity for the general consumer to include it into their diet.

## Figures and Tables

**Table 1:** Variables, factors level, and runs of bee brood protein powder formulation.

Level Code		Variables and Factors Level		
		S_BB Content (g)	GMS Content (g)	CMC Content (g)
−		10	6	0.5
0		20	10	1.0
+		30	14	1.5
Run	Code	S_BB	GMS	CMC
1	(1)	−	−	−
2	a	+	−	−
3	b	−	+	−
4	ab	+	+	−
5	c	−	−	+
6	ac	+	−	+
7	bc	−	+	+
8	abc	+	+	+
9	cp1	0	0	0
10	cp2	0	0	0
11	cp3	0	0	0

(1) = control; a = S_BB content; b = GMS content; c = CMC content; cp = center point; S_BB = steamed bee brood; GMS = glycerol monostearate; CMC = carboxymethyl cellulose.

**Table 2:** Proximate composition of different methods for bee brood preparation.

Parameters	Methods for Bee Brood Preparation		
	Control (Raw Bee Brood)	Boiled Bee Brood (B_BB)	Steamed Bee Brood (S_BB)
Moisture (%) ^ns^	4.61 ± 0.80	3.31 ± 0.28	4.57 ± 0.74
Protein (g/100 g db)	40.40 ± 0.30 ^b^	39.18 ± 0.07 ^c^	44.71 ± 0.07 ^a^
Carbohydrate (g/100 g db)	27.89 ± 0.08 ^b^	32.81 ± 0.17 ^a^	26.92 ± 0.48 ^b^
Lipid (g/100 g db)	29.83 ± 0.33 ^a^	25.32 ± 0.08 ^c^	27.31 ± 0.10 ^b^
Ash (g/100 g db)	4.27 ± 0.01 ^a^	4.07 ± 0.08 ^a^	3.35 ± 0.04 ^b^
Fiber (g/100 g db) ^ns^	2.43 ± 0.04	2.05 ± 0.10	2.50 ± 0.34

Data represented as means ± SD (*n* = 3); a–c Mean values within each row with different superscript letters were significantly different (*p* ≤ 0.05); db = dry basis; ns = not significant.

**Table 3:** Amino acids composition found in different methods of bee brood preparation.

Amino Acids (mg/g db)	Methods for Bee Brood Preparation		
	Control (Raw Bee Brood)	Boiled Bee Brood (B_BB)	Steamed Bee Brood (S_BB)
Aspartic acid	ND	0.58 ± 0.01 ^a^	0.44 ± 0.01 ^b^
Threonine	ND	1.27 ± 0.01 ^b^	1.52 ± 0.01 ^a^
Serine	1.96 ± 0.04 ^a^	0.35 ± 0.01 ^b^	0.28 ± 0.01 ^b^
Glutamic acid	5.96 ± 0.04 ^c^	6.67 ± 0.06 ^a^	6.21 ± 0.04 ^b^
Proline	7.23 ± 0.15 ^a^	5.82 ± 0.13 ^b^	5.30 ± 0.13 ^b^
Glycine	23.87 ± 0.01 ^c^	45.29 ± 0.10 ^a^	34.97 ± 0.11 ^b^
Alanine	16.19 ± 0.16 ^a^	9.34 ± 0.11 ^b^	4.45 ± 0.05 ^c^
Cysteine	1.67 ± 0.01 ^c^	2.13 ± 0.02 ^b^	2.44 ± 0.02 ^a^
Valine	2.48 ± 0.03 ^a^	0.12 ± 0.01 ^b^	0.10 ± 0.01 ^b^
Methionine	0.85 ± 0.01 ^a^	0.76 ± 0.01 ^b^	0.57 ± 0.01 ^c^
Isoleucine	1.22 ± 0.04 ^a^	0.95 ± 0.02 ^b^	0.65 ± 0.01 ^c^
Leucine	1.55 ± 0.02 ^a^	1.43 ± 0.02 ^b^	1.20 ± 0.01 ^c^
Tyrosine	1.05 ± 0.01 ^c^	1.20 ± 0.01 ^b^	1.37 ± 0.01 ^a^
Phenylalanine	1.37 ± 0.01 ^a^	0.98 ± 0.02 ^b^	0.71 ± 0.01 ^c^
Histidine	1.94 ± 0.03 ^c^	4.79 ± 0.03 ^a^	4.43 ± 0.04 ^b^
Lysine	0.31 ± 0.01 ^b^	0.51 ± 0.02 ^a^	0.54 ± 0.01 ^a^
Arginine	ND	0.58 ± 0.01 ^a^	0.44 ± 0.01 ^b^
TAA	67.65 ± 0.57 ^b^	82.19 ± 0.59 ^a^	65.18 ± 0.49 ^c^

Data represented as means ± SD (*n* = 3); a–c mean values within each row with different superscript letters were significantly different (*p* ≤ 0.05); db = dry basis; ND: not detected; TAA = total amino acids indicated sum of 17 amino acids.

**Table 4:** Physicochemical responses for each run of bee brood protein powder, including powder yield, color spaces, water activity, protein content, TAA, and solubility.

Run	Responses							
	Powder Yield (%)	Color Spaces			aw	Protein Content (g/100 g)	TAA (mg/g)	Solubility (%)
		L*	C*	h				
1	5.82 ± 0.01	69.23 ± 0.23	12.01 ± 0.87	65.74 ± 0.98	0.400 ± 0.009	10.77 ± 0.04	13.66 ± 0.11	15.14 ± 0.95
2	8.73 ± 0.01	64.79 ± 0.24	13.93 ± 0.96	66.43 ± 0.09	0.261 ± 0.011	20.35 ± 0.02	22.13 ± 0.12	23.69 ± 0.12
3	10.70 ± 0.02	71.33 ± 0.63	10.01 ± 0.04	59.47 ± 0.88	0.274 ± 0.011	5.31 ± 0.02	12.27 ± 0.07	8.12 ± 0.58
4	10.35 ± 0.01	69.46 ± 0.32	11.51 ± 0.27	63.41 ± 0.18	0.441 ± 0.017	12.88 ± 0.01	17.77 ± 0.08	10.22 ± 0.33
5	6.65 ± 0.02	75.68 ± 0.11	7.71 ± 0.02	58.92 ± 0.35	0.266 ± 0.019	10.16 ± 0.01	9.99 ± 0.10	27.72 ± 0.12
6	10.98 ± 0.01	70.10 ± 0.80	11.43 ± 0.44	63.95 ± 0.25	0.234 ± 0.021	18.08 ± 0.02	20.14 ± 0.08	17.25 ± 0.27
7	9.74 ± 0.02	77.66 ± 0.08	8.84 ± 0.11	58.03 ± 0.21	0.290 ± 0.010	5.06 ± 0.03	6.87 ± 0.0.6	16.71 ± 0.41
8	11.30 ± 0.02	75.07 ± 0.14	8.11 ± 0.92	60.77 ± 0.03	0.365 ± 0.014	11.39 ± 0.02	13.58 ± 0.03	18.74 ± 0.86
9	9.41 ± 0.01	70.79 ± 0.01	11.32 ± 0.01	63.31 ± 0.23	0.229 ± 0.013	13.32 ± 0.01	15.64 ± 0.12	18.63 ± 0.76
10	9.01 ± 0.01	69.18 ± 0.39	11.70 ± 0.85	64.39 ± 0.90	0.238 ± 0.020	13.40 ± 0.01	14.56 ± 0.11	19.41 ± 0.43
11	9.68 ± 0.01	73.88 ± 0.04	11.41 ± 0.02	65.23 ± 0.02	0.269 ± 0.018	12.79 ± 0.02	13.29 ± 0.04	17.86 ± 0.91

All data are expressed as the mean ± SD of triplicate measurements; aw = water activity; TAA = total amino acids indicated sum of 17 amino acids.

**Table 5:** Equation models, adjusted R^2^, and coefficient of variation (C.V.) for the selected dependent variable responses for bee brood protein powder production.

Responses	Equations	Adjusted R^2^	C.V. (%)
Powder yield (%)	=−0.70 + 0.21A + 0.88B + 1.03C − 0.02AB + 0.08AC − 0.19BC	0.9767	2.83
L*	=67.62 − 0.31A + 0.07B + 6.75C + 0.02AB − 0.05AC + 0.01BC	0.7841	2.40
h	=67.11 + 0.15A − 0.42B − 3.35C	0.6444	2.77
Protein content (g/100 g)	=13.17 + 0.39A − 0.77B − 1.16C	0.9491	8.51
TAA (mg/g)	=15.46 + 0.39A − 0.48B − 3.81C	0.9173	8.59

A = S_BB content (g); B = GMS content (g); C = CMC content (g).

## Data Availability

The original contributions presented in the study are included in the article, further inquiries can be directed to the corresponding author.

## References

[1] Finke M.D. (2005). Nutrient Composition of Bee Brood and its Potential as Human Food. Ecol. Food Nutr..

[2] Ghosh S., Jung C., Meyer-Rochow V.B. (2016). Nutritional value and chemical composition of larvae, pupae, and adults of worker honey bee, *Apis mellifera ligustica* as a sustainable food source. J. Asia-Pac. Entomol..

[3] Lecocq A., Foley K., Jensen A.B. (2018). Drone brood production in Danish apiaries and its potential for human consumption. J. Apic. Res..

[4] Ghosh S., Meyer-Rochow V.B., Jung C. (2021). Honey bees and their brood: A potentially valuable resource of food, worthy of greater appreciation and scientific attention. J. Ecol. Environ..

[5] Jensen A.B., Evans J., Jonas-Levi A., Benjamin O., Martinez I., Dahle B., Roos N., Lecocq A., Foley K. (2019). Standard methods for *Apis mellifera* brood as human food. J. Apic. Res..

[6] Van Huis A. (2013). Potential of insects as food and feed in assuring food security. Annu. Rev. Entomol..

[7] Liceaga A.M. (2021). Processing insects for use in the food and feed industry. Curr. Opin. Insect. Sci..

[8] Mishyna M., Martinez J.J.I., Chen J., Benjamin O. (2019). Extraction, characterization and functional properties of soluble proteins from edible grasshopper (*Schistocerca gregaria*) and honey bee (*Apis mellifera*). Food Res. Int..

[9] Hardy Z., Jideani V.A. (2017). Foam-mat drying technology: A review. Crit. Rev. Food Sci. Nutr..

[10] Sangamithra A., Sivakumar V., John S.G., Kannan K. (2015). Foam mat drying of food materials: A review. J. Food Process. Preserv..

[11] Kamali R., Dadashi S., Dehghannya J., Ghaffari H. (2022). Numerical simulation and experimental investigation of foam-mat drying for producing banana powder as influenced by foam thickness. Appl. Food Res..

[12] Warepam S.C., Jena S. (2022). Optimization of pomelo (*Citrus grandis* L. *Osbeck*) juice foam composition: Effect of foam composition on foam quality. J. Food Process. Preserv..

[13] Reis F.R., de Moraes A.C.S., Masson M.L. (2021). Impact of Foam-Mat Drying on Plant-Based Foods Bioactive Compounds: A Review. Plant Foods Hum. Nutr..

[14] Association of International Official Analytic Chemists (AOAC) (2010). Official Methods of Analysis.

[15] Somjai C., Siriwoharn T., Kulprachakarn K., Chaipoot S., Phongphisutthinant R., Chaiyana W., Srinuanpan S., Wiriyacharee P. (2022). Effect of drying process and long-term storage on characterization of Longan pulps and their biological aspects: Antioxidant and cholinesterase inhibition activities. LWT.

[16] Thakur C., Verma A.K., Sharma P.C., Kaushal M., Vaidya D., Sharma R.C., Shivani (2021). Effect of foaming agents on foaming properties, drying time and powder yield of rainy season *Psidium guajava* fruits cv. Shweta. J. Pharm. Innov..

[17] Vidović S.S., Vladić J.Z., Vaštag Ž.G., Zeković Z.P., Popović L.M. (2014). Maltodextrin as a carrier of health benefit compounds in Satureja montana dry powder extract obtained by spray drying technique. Powder Technol..

[18] Omidi S., Aarabi A., Zaki Dizaji H., Shahdadi F. (2024). Microwave-assisted foam mat drying of red beet pulp: Influence of milk protein concentrate (MPC) and maltodextrin as a foaming agent, optimization and quality attribute. J. Food Meas. Charact..

[19] Melgar-Lalanne G., Hernández-Álvarez A.J., Salinas-Castro A. (2019). Edible Insects Processing: Traditional and Innovative Technologies. Compr. Rev. Food Sci. Food Saf..

[20] Ojha S., Bußler S., Psarianos M., Rossi G., Schlüter O.K. (2021). Edible insect processing pathways and implementation of emerging technologies. J. Insects Food Feed..

[21] Nyangena D.N., Mutungi C., Imathiu S., Kinyuru J., Affognon H., Ekesi S., Nakimbugwe D., Fiaboe K.K.M. (2020). Effects of Traditional Processing Techniques on the Nutritional and Microbiological Quality of Four Edible Insect Species Used for Food and Feed in East Africa. Foods.

[22] Djikeng T.F., Mouto Ndambwe C.M., Ngangoum E.S., Tiencheu B., Tambo Tene S., Achidi A.U., Womeni H.M. (2022). Effect of different processing methods on the proximate composition, mineral content and functional properties of snail (*Archachatina marginata*) meat. J. Agric. Food Res..

[23] Manditsera F.A., Luning P.A., Fogliano V., Lakemond C.M.M. (2019). Effect of domestic cooking methods on protein digestibility and mineral bioaccessibility of wild harvested adult edible insects. Food Res. Int..

[24] Parkinson Markmanuel D., Godwin J. (2020). Effects of Culinary Methods on The Proximate Composition of an Edible Insect (Rhynchophorus Phoenicis) Larvae Obtained from Bayelsa State, Nigeria. Eur. J. Agric. Food Sci..

[25] Somjai C., Siriwoharn T., Kulprachakarn K., Chaipoot S., Phongphisutthinant R., Wiriyacharee P. (2021). Utilization of Maillard reaction in moist-dry-heating system to enhance physicochemical and antioxidative properties of dried whole longan fruit. Heliyon.

[26] de Cól C.D., Tischer B., Hickmann Flôres S., Rech R. (2021). Foam-mat drying of bacaba (*Oenocarpus bacaba*): Process characterization, physicochemical properties, and antioxidant activity. Food Bioprod. Process..

[27] Mohamed A.A., Ismail-Fitry M.R., Rozzamri A., Bakar J. (2022). Effect of foam-mat drying on kinetics and physical properties of Japanese threadfin bream (*Nemipterus japonicus*) powder. J. Food Process. Preserv..

[28] Bhardwaj M., Sharma P.C., Verma A., Thakur C., Saini R., Shivani (2020). Improving the Powder Yield and Foaming Characteristics of Papaya Leaf Juice Treated with CMC (Carboxy-Methyl-Cellulose) and GMS (Glycerol-Mono-Stearate). Int. J. Curr. Microbiol. Appl. Sci..

[29] Brar A.S., Kaur P., Kaur G., Subramanian J., Kumar D., Singh A. (2020). Optimization of Process Parameters for Foam-Mat Drying of Peaches. Int. J. Fruit Sci..

[30] Silva J.M., Crozatti T.T.d.S., Saqueti B.H.F., Chiavelli L.U.R., Matioli G., Santos O.O. (2024). Optimization of foam mat drying using Central Composite Design to produce mixed juice powder: A process and characterization study. Food Bioprod. Process..

[31] Bahriye G., Dadashi S., Dehghannya J., Ghaffari H. (2023). Influence of processing temperature on production of red beetroot powder as a natural red colorant using foam-mat drying: Experimental and modeling study. Food Sci. Nutr..

